# Profound Vision Loss With Minimal Retinal Findings: A Case of Microvascular Ischemia Unraveled by Multimodal Imaging

**DOI:** 10.7759/cureus.90605

**Published:** 2025-08-20

**Authors:** Petros Asteris, Georgios Stergiopoulos, Eleni Bagli, Chris Kalogeropoulos

**Affiliations:** 1 Department of Ophthalmology, University Hospital of Ioannina, Ioannina, GRC

**Keywords:** atrial fibrillation (af), central retinal vein occlusion (crvo), faf, multimodal ophthalmic imaging, oct angiography, pamm

## Abstract

An 80-year-old woman presented with sudden, profound vision loss in her left eye. Initial fundus examination showed only mild disc hyperemia, a few retinal hemorrhages, and venous dilation, findings insufficient to explain the severity of visual loss on their own. Optical coherence tomography (OCT) revealed hyperreflective bands in the inner nuclear layer consistent with paracentral acute middle maculopathy (PAMM), while en face OCT angiography demonstrated a fern-like perivenular pattern. A relative afferent pupillary defect (RAPD) was also noted at the 48-hour follow-up. Given the clinical-imaging discrepancy, fluorescein angiography, performed at the 48-hour follow-up, showed delayed arterial and venous filling, macular capillary dropout, and late optic disc leakage. Fundus autofluorescence revealed marked hypoautofluorescence in ischemic regions. Despite initial apparent anatomical resolution, vision remained poor, and the RAPD persisted. On the last follow-up, retinal atrophy had developed. Systemic evaluation identified previously undiagnosed atrial fibrillation, implicating microembolic retinal ischemia as the likely etiology. No emboli were visualized, but imaging suggested occlusion of small-caliber retinal vessels. No visual recovery was observed at follow-ups. To our knowledge, reports linking fern-like PAMM to newly discovered atrial fibrillation with this degree of vision loss are uncommon. This case underscores that profound vision loss may result from microvascular ischemia in the absence of overt fundus signs. It highlights the importance of multimodal imaging and cardiovascular assessment in unexplained acute visual loss.

## Introduction

Paracentral acute middle maculopathy (PAMM) is an optical coherence tomography (OCT)-defined retinal finding characterized by hyperreflective bands in the inner nuclear layer (INL), typically reflecting ischemia of the intermediate and deep capillary plexuses [1]. It is most often associated with transient or mild vision disturbances, such as paracentral scotomas [2]. It is most frequently observed in retinal vascular disorders, including vein and artery occlusions, diabetic retinopathy, and hypertensive retinopathy [2]. In most cases, PAMM signifies a localized, non-catastrophic ischemic event [2].

Profound vision loss in the context of PAMM is uncommon [2] and often signals major vascular or significant underlying microvascular compromise that may not be evident on initial funduscopic examination [2-4]. In some cases, subtle or minimal retinal findings can misrepresent significant ischemic injury at the capillary level. These presentations may be driven by microvascular embolic events that escape clinical detection, particularly in patients with systemic embolic risk factors such as atrial fibrillation [2]. As a result, the true extent of ischemia may only be revealed through advanced multimodal imaging and systemic evaluation.

We report a rare case of acute, severe monocular vision loss in an elderly woman with minimal funduscopic findings and no visible emboli. Multimodal retinal imaging revealed a fern-like PAMM pattern, with inner retinal infarction and macular capillary dropout. Subsequent systemic evaluation uncovered previously undiagnosed atrial fibrillation, strongly implicating an underlying microembolic mechanism. To the best of our knowledge, there are only a few reports associating fern-like PAMM with undiagnosed atrial fibrillation and such severe vision loss.

This case underscores the importance of considering microembolic etiologies in patients with unexplained retinal ischemia with minimal fundus findings. It highlights the diagnostic value of advanced imaging and systemic workup in revealing hidden but vision-threatening pathology.

## Case presentation

An 80-year-old woman was referred to the emergency department of our clinic due to acute vision loss in her left eye. The patient arrived at our department a total of five hours after the onset of vision loss. The patient was receiving treatment for hypertension and dyslipidemia. The rest of her known medical history did not reveal any additional information.

**Figure 1:**
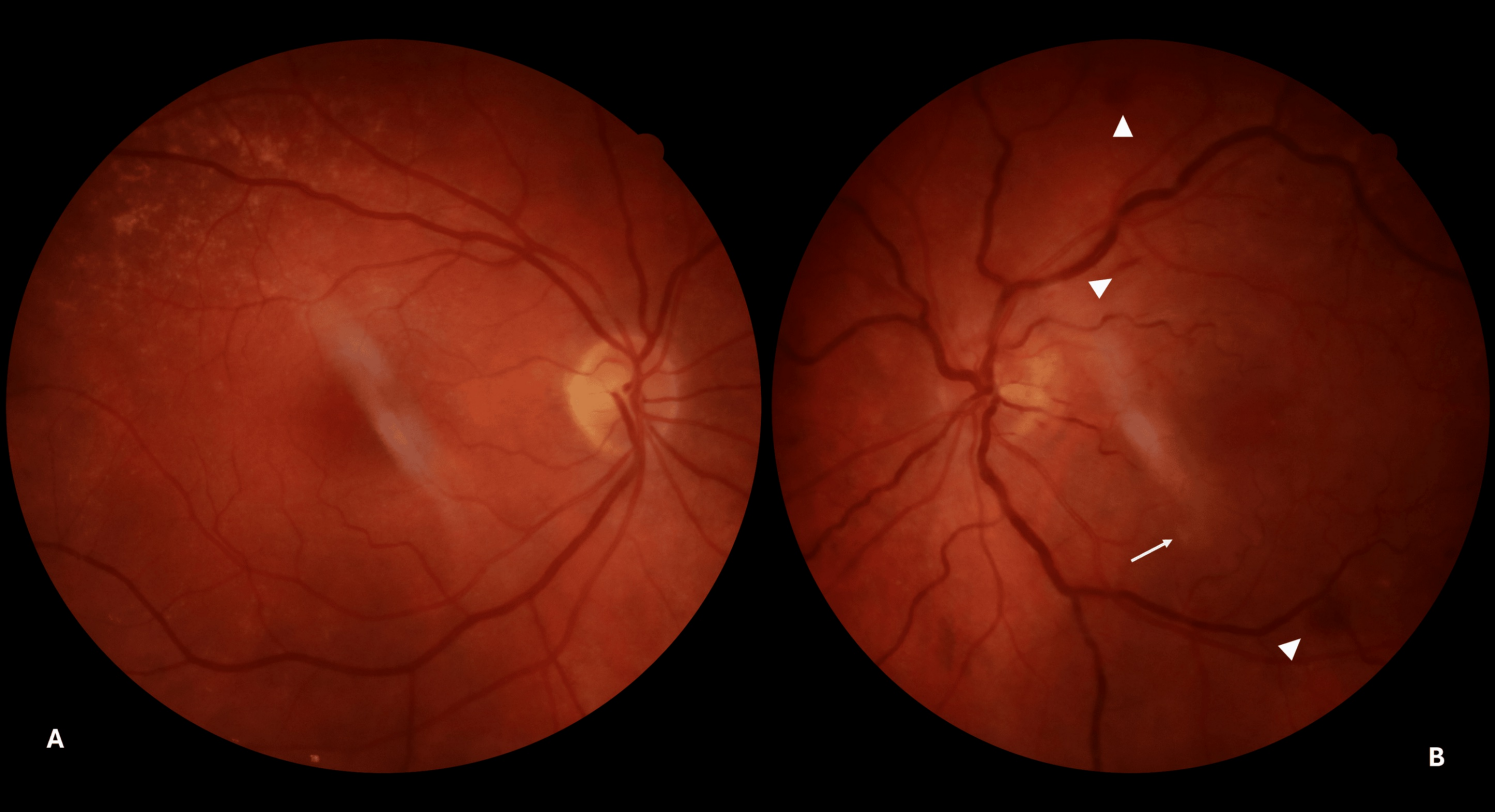
Day 0 fundus photographs at presentation (A) Right eye: normal optic disc, macula, and retinal vasculature. (B) Left eye: mild disc hyperemia with engorged retinal veins; three discrete hemorrhages (white arrowheads) and a faint grey-white paracentral band at the macula (white arrow) are annotated, findings that appear disproportionately mild relative to the patient’s counting-fingers vision. Images acquired using Canon CR2 Digital Fundus Camera (Canon Inc., Ōta, Tokyo).

A full routine examination was subsequently performed. The patient had a visual acuity (VA) of 6/7.5 Snellen for her right eye and counting fingers for her left eye. A previous examination by the referring ophthalmologist, six months before this incident, reported a VA of 6/7.5 Snellen for both eyes. The right pupillary reaction was normal, while the left pupil was in pharmaceutical mydriasis. Intraocular pressure, measured with Goldmann applanation tonometry, was 15 mmHg and 16 mmHg for the right and left eyes, respectively. Both eyes were pseudophakic, with posterior chamber intraocular lenses. No additional findings were noted from the examination of the anterior segment. Subsequently, a dilated fundus examination was conducted for both eyes (Figure 1).

The fundus of the right eye was normal (Figure 1A). The fundus of the left eye revealed mild disc hyperemia with a limited number of retinal hemorrhages (flame-shaped and dot/blot), most at the posterior pole and a few at the periphery. Enlarged and tortuous retinal veins were noted. Neither a boxcarring appearance of the retinal arteries nor a cherry-red macula with retinal edema was observed (Figure 1B).

**Figure 2:**
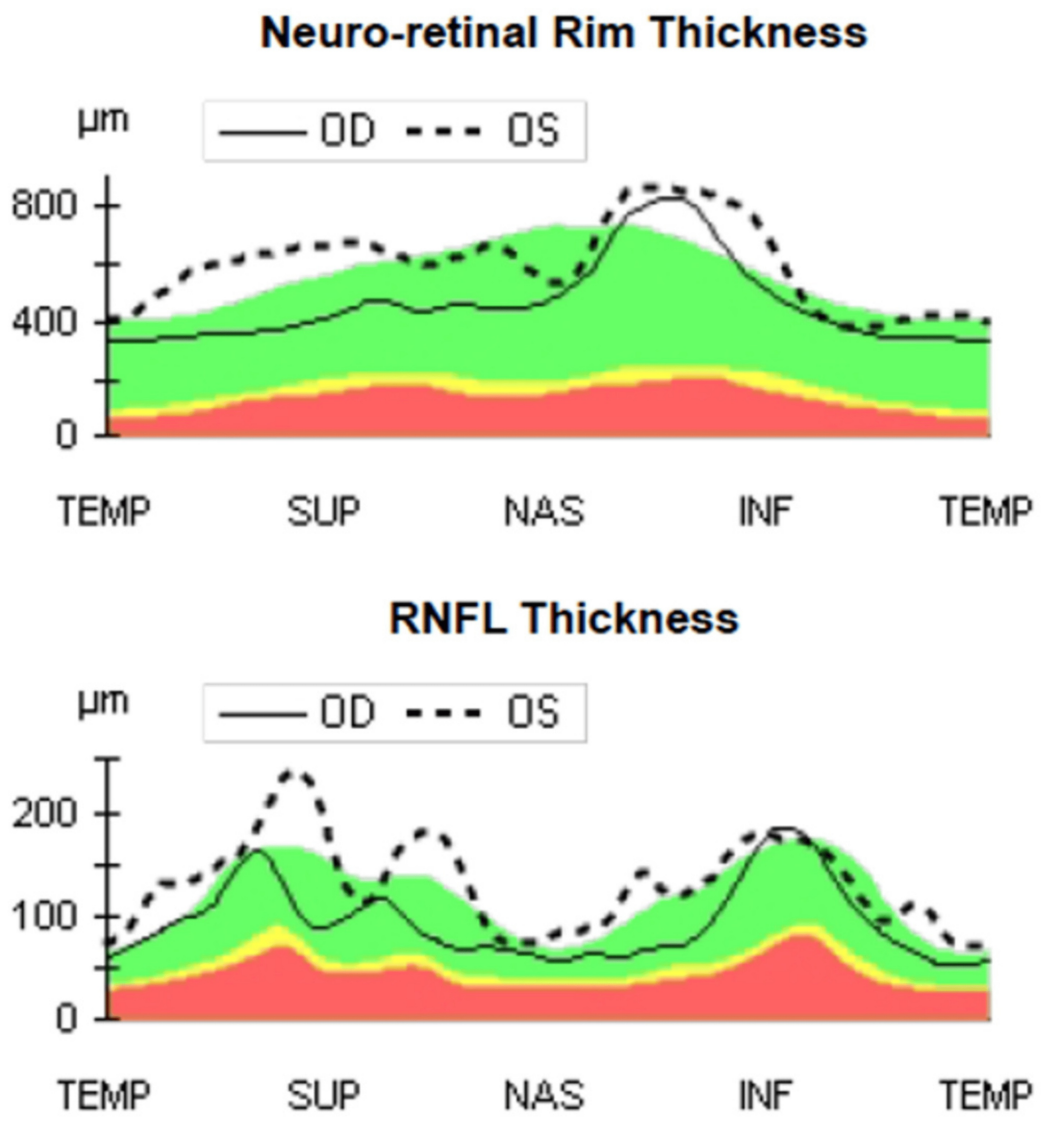
Day 0 spectral-domain OCT scan comparing the fellow right eye (solid line) with the affected left eye (dashed line) (Top) neuro-retinal rim thickness. (Bottom) RNFL thickness. The left eye shows diffuse, mild thickening (most marked in the superior and inferior sectors) while remaining largely within normative limits (green band). The shaded background represents normative data ranges: green = within normal limits, yellow = borderline, and red = outside normal limits. Images acquired using Zeiss Cirrus 5000 OCT (Carl Zeiss Meditec AG, Baden-Württemberg, Germany). OCT: optical coherence tomography, RNFL: retinal nerve fiber layer, TEMP: temporal part of optic nerve head, SUP: superior part of optic nerve head, NAS: nasal part of optic nerve head, INF: inferior part of optic nerve head, OD: right eye, OS: left eye

Following these findings, OCT of the optic disc and macula and OCT-angiography (OCT-A) of the macula were performed in both eyes. Retinal nerve fiber layer (RNFL) and optic disc rim thickness were within normal values for the right eye and slightly elevated for the left eye (Figure 2).

**Figure 3:**
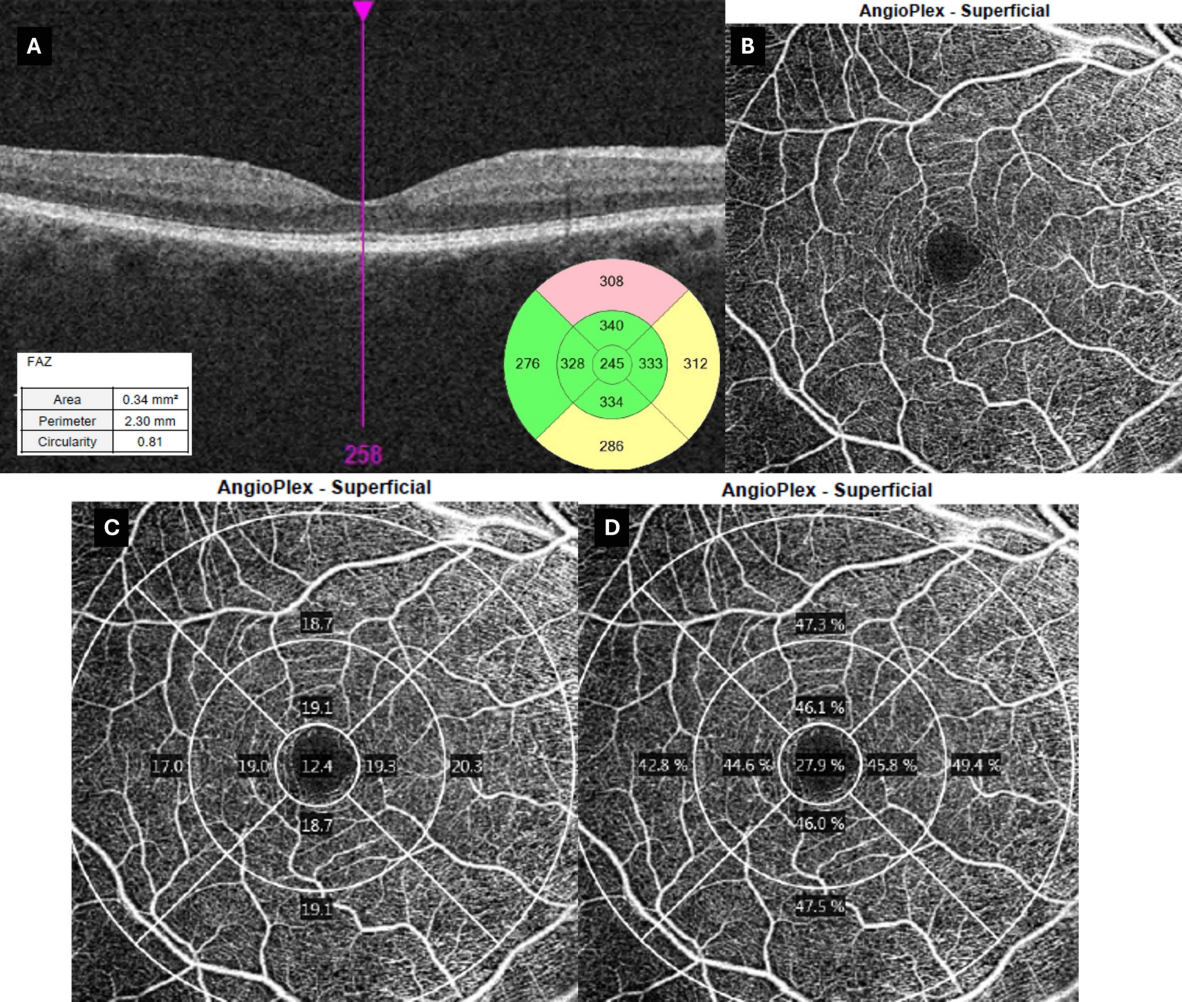
Day 0 multimodal OCT/OCT-A (6 × 6 mm) of the fellow (right) eye (A) Horizontal spectral-domain OCT B-scan with ETDRS thickness map shows normal foveal contour and thickness. Inset: FAZ metrics are within normal limits (area 0.34 mm²; circularity 0.81). (B) 6 × 6 mm superficial angiogram demonstrates an intact perifoveal capillary arcade. (C) Vessel density plot. (D) Perfusion density plot. Both plots reveal homogeneously preserved flow with only minimal percentage deviation from the device’s normative database. The unaffected right eye serves as an internal control, emphasizing the unilateral, ischemic nature of the pathology documented in the left eye. Images acquired using Zeiss Cirrus 5000 OCT (Carl Zeiss Meditec AG, Baden-Württemberg, Germany). OCT: optical coherence tomography, OCT-A: optical coherence tomography-angiography, ETDRS: early treatment of diabetic retinopathy study, FAZ: foveal avascular zone

No findings were observed in the macula in the patient’s right eye (Figure 3).

**Figure 4:**
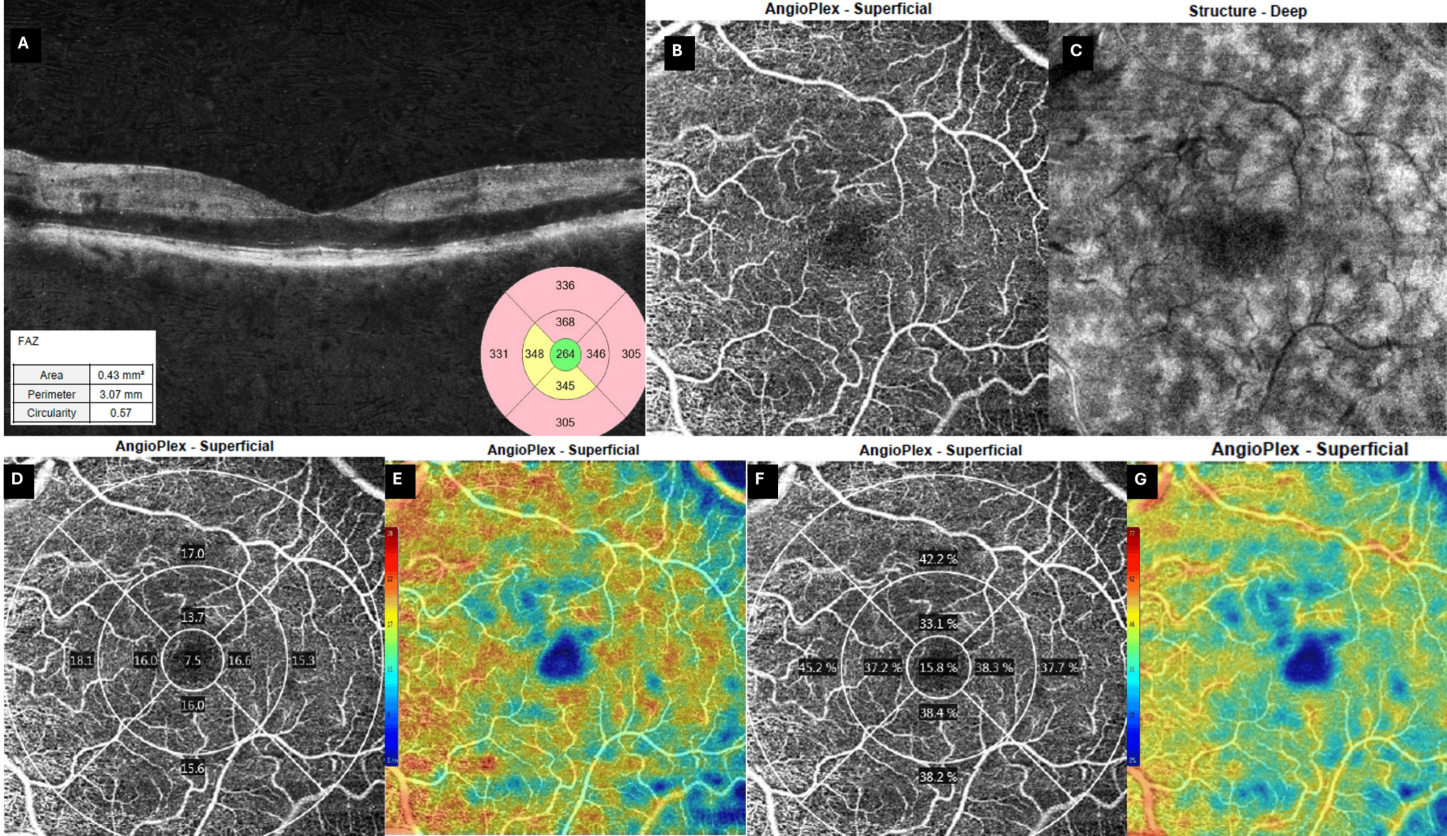
Day 0 multimodal OCT and OCT-A (6 × 6 mm) of the affected (left) eye (A) Horizontal B-scan shows placoid hyper-reflective bands in the INL. FAZ is enlarged (0.43 mm²) and irregular (circularity 0.57). (B) OCT-A of the superficial plexus shows compromise of vessel architecture, with capillary attenuation and loss, compatible with ischemic insult. (C) Deep-structure slab highlights a fern-like perivenular PAMM pattern. (D) Vessel-density and (F) perfusion-density ETDRS plots show marked flow reduction, especially in the central 1-mm ring (7.5% and 15.8%, respectively) and the second ring. (E, G) Corresponding color-coded deviation maps of vascular density and perfusion density (respectively) depict a hypoperfused macula (scale ranging from blue/low to red/high density). Collectively, the images confirm acute deep-capillary ischemia with a perivenular distribution. Images acquired using Zeiss Cirrus 5000 OCT (Carl Zeiss Meditec AG, Baden-Württemberg, Germany). OCT: optical coherence tomography, OCT-A: optical coherence tomography-angiography, ETDRS: early treatment of diabetic retinopathy study, FAZ: foveal avascular zone, INL: inner nuclear layer

OCT of the left eye did not reveal any macular edema. However, it did reveal the presence of placoid, hyperreflective bands at the level of the INL, sparing the outer retina (Figure 4A), and a compromised superficial vascular plexus (Figure 4B). At this time, these findings were consistent with the presence of PAMM, with a fern-like pattern revealed on en face OCT-A (Figure 4C), possibly on the grounds of a transient or partial central retinal vein occlusion (CRVO). However, the severity of symptoms was disproportionate to these findings and prompted further investigation for an ischemic cause. OCT-A vascular density and perfusion were notably reduced compared to the fellow eye (Figure 4D-4G).

A laboratory workup was conducted to exclude the presence of a new acute condition, most notably giant cell arteritis (GCA) (ESR, CRP, and platelet count). No noteworthy results indicative of an acute condition were observed on this first investigative lab workup. Further laboratory workup was ordered at the two-day follow-up, which came back negative for any new systemic ailments or deregulation of known hyperlipidemia (Appendices). CT of the brain revealed only cerebral atrophy with some enlargement of sulci and ventricles, consistent with patient age.

**Figure 5:**
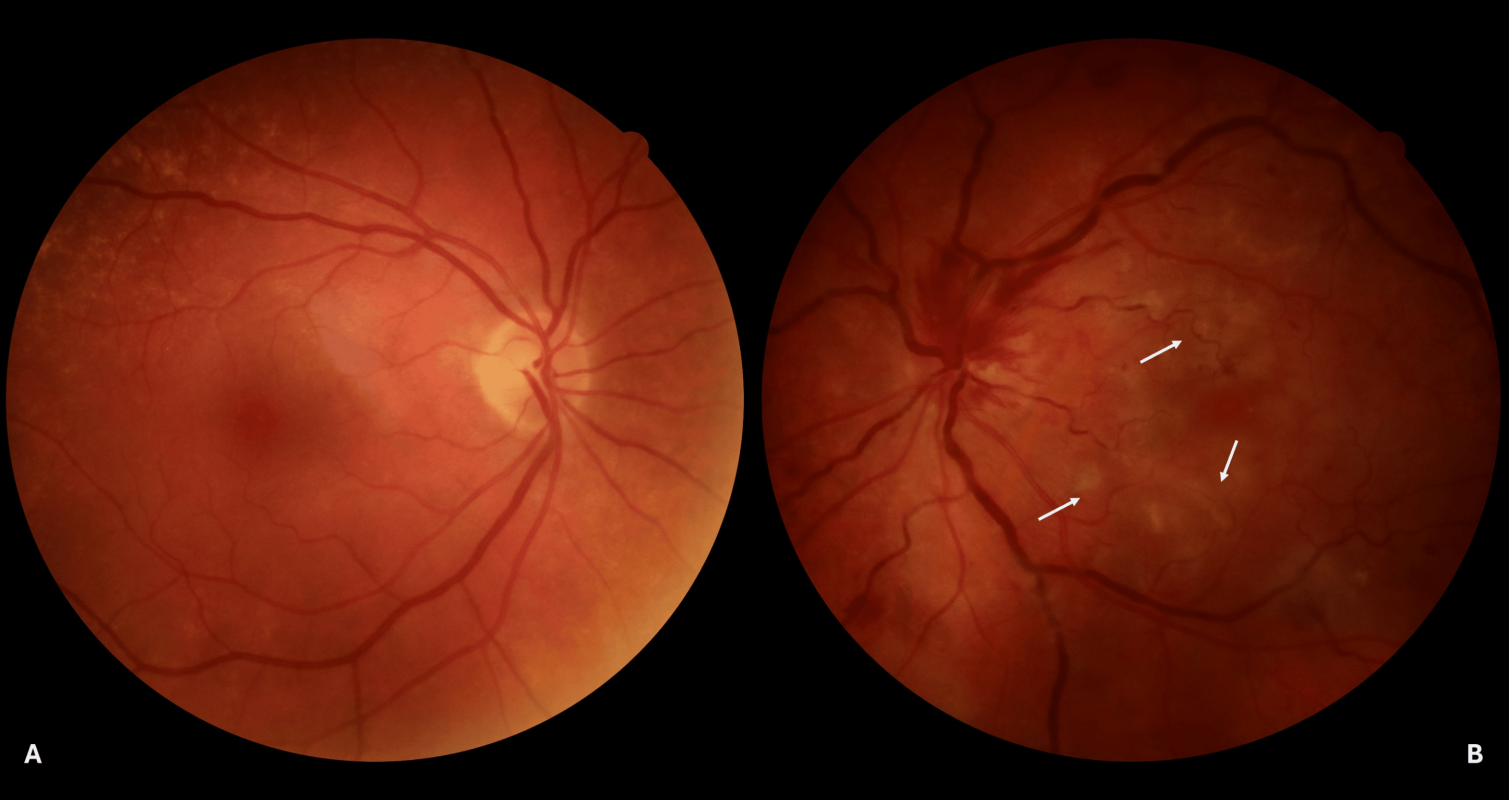
Day 2 color fundus photographs (A) Unaffected right eye remains normal. (B) Left eye demonstrates intensified optic-disc hyperemia with new flame-shaped hemorrhages (mostly superotemporal) and venous engorgement. Several grey-white placoid lesions are now visible in the macula (white arrows), corresponding to the fern-like PAMM pattern seen on OCT-A. The interval evolution illustrates the progression of deep-capillary ischemia accompanied by limited secondary venous stasis, rather than a classic ischemic CRVO. Images acquired using Canon CR2 Digital Fundus Camera (Canon Inc., Ōta, Tokyo). OCT-A: optical coherence tomography-angiography, PAMM: paracentral acute middle maculopathy, CRVO: central retinal vein occlusion

The patient did not wish to be hospitalized. A fundus fluorescein angiography (FA) was scheduled and performed as soon as the patient was available, two days after the first examination. At the two-day follow-up, the anterior segment examination did not reveal any pathological findings except for a relative afferent pupillary defect (RAPD) in the left eye. During fundoscopy, the fellow eye was still normal (Figure 5A). More retinal hemorrhages had appeared, mostly flame-shaped, at the optic disc in the left eye, along with white deep retina lesions at the macula (Figure 5B).

**Figure 6:**
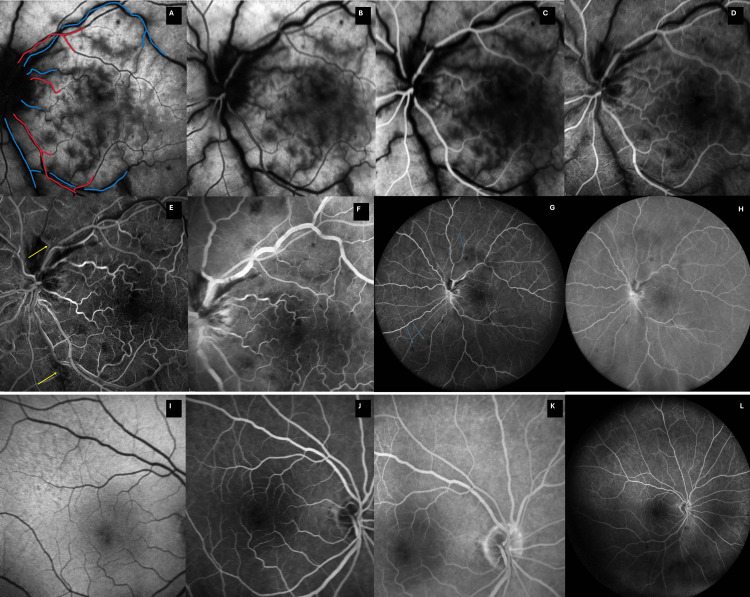
Day 2 FAF and FA images for the left eye (A-H) and the right eye (I-L) (A) FAF shows confluent hypoautofluorescence in a fern-like perivenular pattern, mirroring the OCT-A PAMM map and indicating severe inner-retinal infarction. Findings are too profound for classic perivenular PAMM. Arteries are annotated in red and veins in blue. (B) Early arterial FA, delayed arm-to-retina time (17 seconds). (C) Late arterial phase FA (21 seconds), delayed transition to venous phase. (D) Early venous phase FA (30 seconds). (E) Mid-phase FA (35 seconds) reveals an enlarged, irregular FAZ with capillary dropout. Two venular branches with delayed filling are annotated (yellow arrows). Some low-perfusion areas are also noted. (F) Late-phase FA (11 minutes) confirms persistent perifoveal non-perfusion and FAZ irregularity. Late disc leakage is present. (G, H) Wide-field FA, early and late frames (respectively), confirm macular-centered non-perfusion without peripheral ischemia. Blue arrows show some deep retinal hemorrhages. (I–L) Control right-eye FAF and FA frames are entirely normal. Images acquired using Heidelberg SPECTRALIS (Heidelberg Engineering GmbH, Heidelberg, Germany). FA: fundus fluorescein angiography, FAF: fundus autofluorescence, OCT-A: optical coherence tomography-angiography, PAMM: paracentral acute middle maculopathy, FAZ: foveal avascular zone

Fundus autofluorescence (FAF) and FA of both eyes were subsequently performed (Figure 6).

FAF of the affected areas was strongly hypoautofluorescent (Figure 6A). These areas also corresponded to white, possibly ischemic regions involving the deep retina and were observed on fundoscopy but were better visualized on fundus photography (Figure 5B). These correspond to previously observed areas of PAMM.

**Figure 7:**
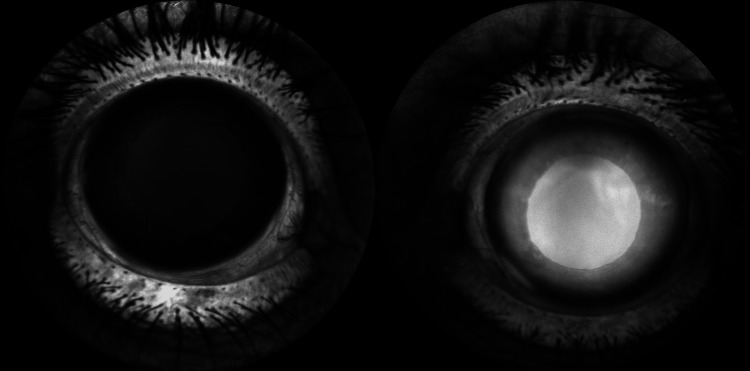
Day 2 late-phase, anterior-segment FA Left: unaffected right eye shows crisp iris vessels with only faint physiological staining. Right: affected left eye demonstrates diffuse, homogeneous hyperfluorescence of the iris and pupillary zone, indicating marked vascular leakage. Images acquired on Heidelberg SPECTRALIS (Heidelberg Engineering GmbH, Heidelberg, Germany). FA: fundus fluorescein angiography

FA of the left eye revealed delayed arm-to-retina time (Figure 6B), a slightly delayed but even filling of retinal arteries (Figure 6C), delayed transition to the venous phase (Figure 6D), and delayed filling of two retinal vein branches compared to the rest of the venular retinal vasculature (Figure 6E). FA also revealed enlarged and irregular FAZ and increased intercapillary spacing of macular vascular plexus (Figure 6E-6F). Late fluorescein leakage at the optic disc was also observed (Figure 6F). No peripheral ischemia was noted (Figure 6G-6H). No noteworthy findings were observed in the right eye (Figure 6I-6L). Diffuse, late iris leakage during FA was also noted in the left eye (Figure 7).

**Figure 8:**
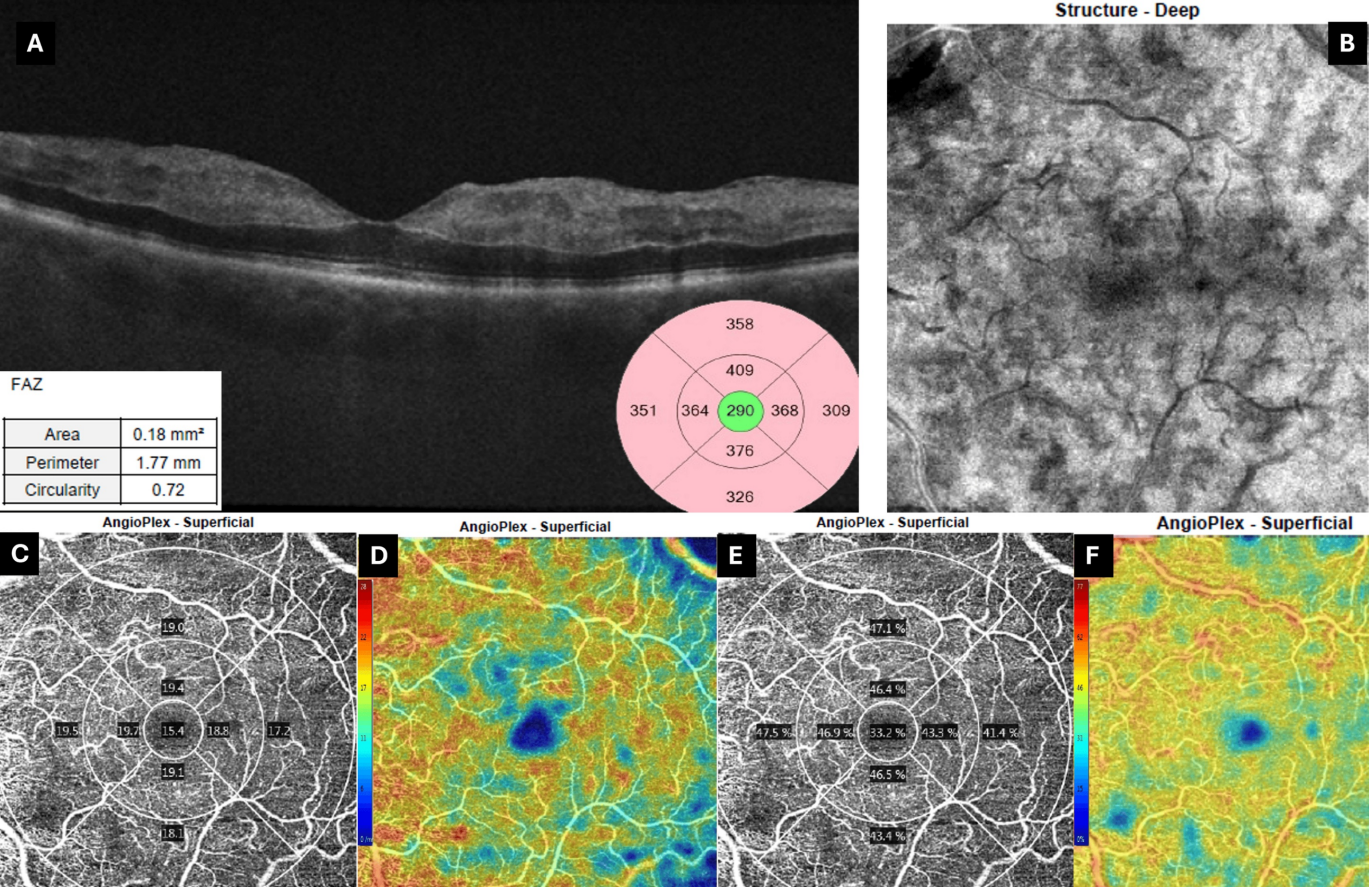
Day 2 macular OCT and OCT-A (6 × 6 mm) of the affected eye (A) Spectral-domain OCT shows persistent inner-nuclear-layer hyper-reflectivity with mild diffuse thickening. Hyperreflectivity expands and involves more retinal layers. FAZ metrics (area 0.18 mm²; circularity 0.72) appear markedly improved. (B) Deep-structure en-face image demonstrates progression of the fern-like ischemic pattern. (C, E) ETDRS vessel-density and perfusion-density plots, respectively, reveal a striking, though likely artifactual, rise in macular flow metrics from the day-0 examination (center 15.4%/33.2%), exceeding even normal eye metrics. (D, F) Corresponding color-coded deviation maps show a warmer-colored macula, with seemingly increased vascular density and perfusion (scale ranging from blue/low to red/high density). Images acquired using Zeiss Cirrus 5000 OCT (Carl Zeiss Meditec AG, Baden-Württemberg, Germany). OCT: optical coherence tomography, OCT-A: optical coherence tomography-angiography, FAZ: foveal avascular zone, ETDRS: early treatment of diabetic retinopathy study

Follow-up OCT revealed extensive hyperreflectivity at the level of the INL (Figure 8A), extending to the inner retina. This was accompanied by extension of the fern-like pattern on en face OCT-A (Figure 8B). An extensive increase in vascular density and perfusion density was noted on OCT-A at this time (Figure 8C-8F). These values exceeded the metrics of the fellow normal eye (Figure 3), which was paradoxical and likely artifactual/reperfusion related.

The patient was scheduled for an MRI of the eye and orbit, which was unremarkable; a complete examination by a cardiologist and neurologist; and a color duplex ultrasound of the carotid and vertebral arteries to assess any blood flow restrictions and plaque formation. Although most systemic findings were age-appropriate, cardiovascular evaluation during the first week revealed previously undiagnosed atrial fibrillation. The patient was administered oral rivaroxaban 20 mg once daily for the reduction of embolic risk and bisoprolol starting at 2.5 mg once daily for the control of cardiac rhythm.

**Figure 9:**
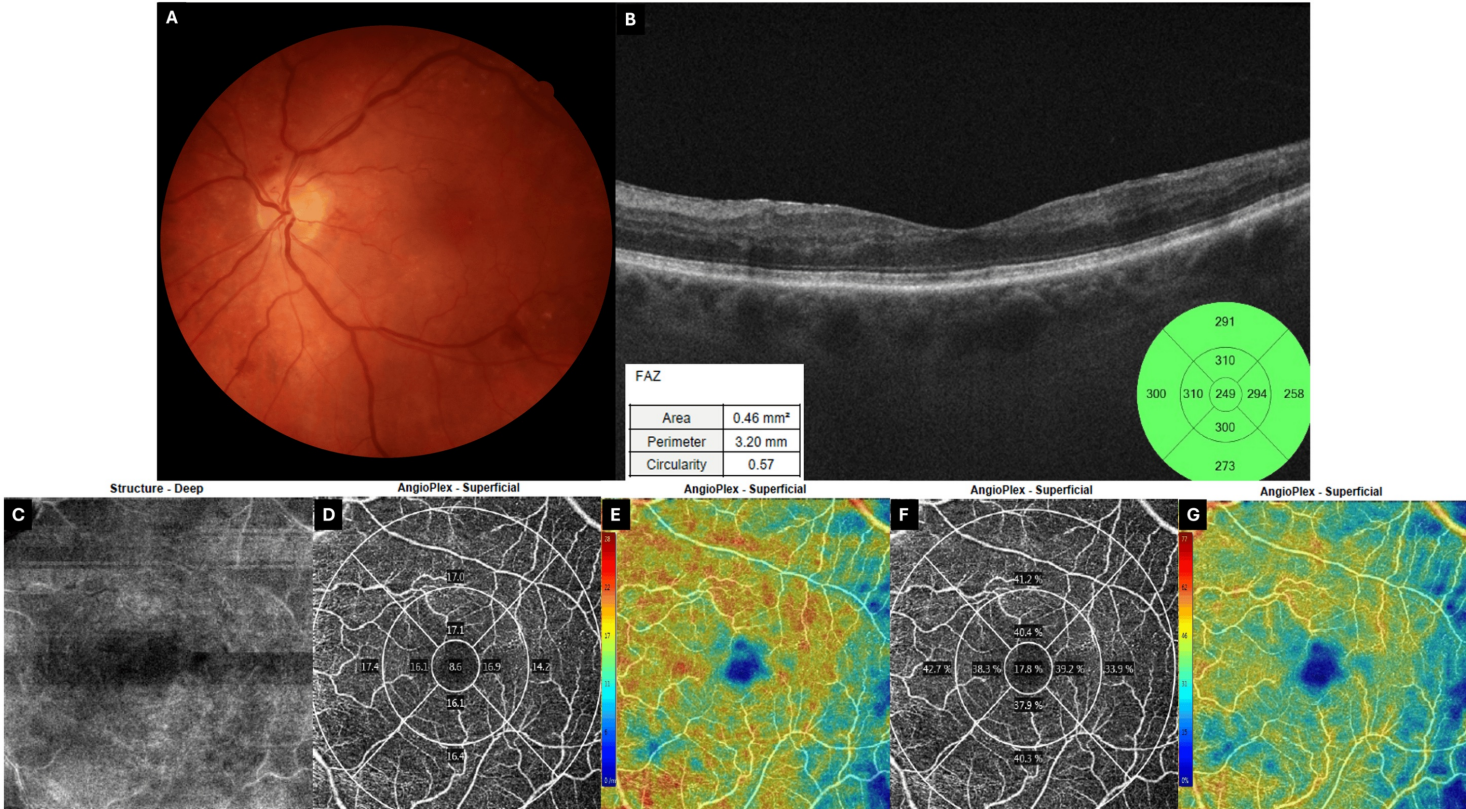
One-month follow-up imaging of the affected (left) eye (A) Color fundus photograph shows resolution of most hemorrhages and disc hyperemia, leaving a flat, pale macula. (B) Spectral-domain OCT reveals inner-retinal thinning at the fovea (center 249 µm). FAZ is now enlarged (0.46 mm²) and irregular (circularity 0.57), reflecting permanent capillary loss. PAMM lesions are still present but in regression. (C) Deep-structure en-face image depicts the original fern-like PAMM in regression, without the previous evident pattern. (D, F) ETDRS vessel- and perfusion-density plots (respectively) show a marked drop in central macular flow (8.6%/17.8%), reversing the transient hyperdensity seen at the 48-hour examination. (E, G) Corresponding deviation maps display a broad blue hypoperfused macular zone (scale ranging from blue/low to red/high density). OCT and OCT-A (6 × 6 mm) images acquired using Zeiss Cirrus 5000 OCT (Carl Zeiss Meditec AG, Baden-Württemberg, Germany). Fundus images acquired using Canon CR2 Digital Fundus Camera (Canon Inc., Ōta, Tokyo). OCT: optical coherence tomography, FAZ: foveal avascular zone, PAMM: paracentral acute middle maculopathy, ETDRS: early treatment of diabetic retinopathy study

A follow-up examination was scheduled at one and three months post presentation. At the one-month follow-up, most imaging and fundoscopic findings had started to resolve (Figure 9), but the patient had not recovered any vision. Macular thickness was at this time within normal values, and hyperreflective lesions had dissipated to some degree (Figure 9B). Macular edema was not observed. OCT-A vascular density and perfusion returned to the values of the first examination (Figure 9D-9G).

**Figure 10:**
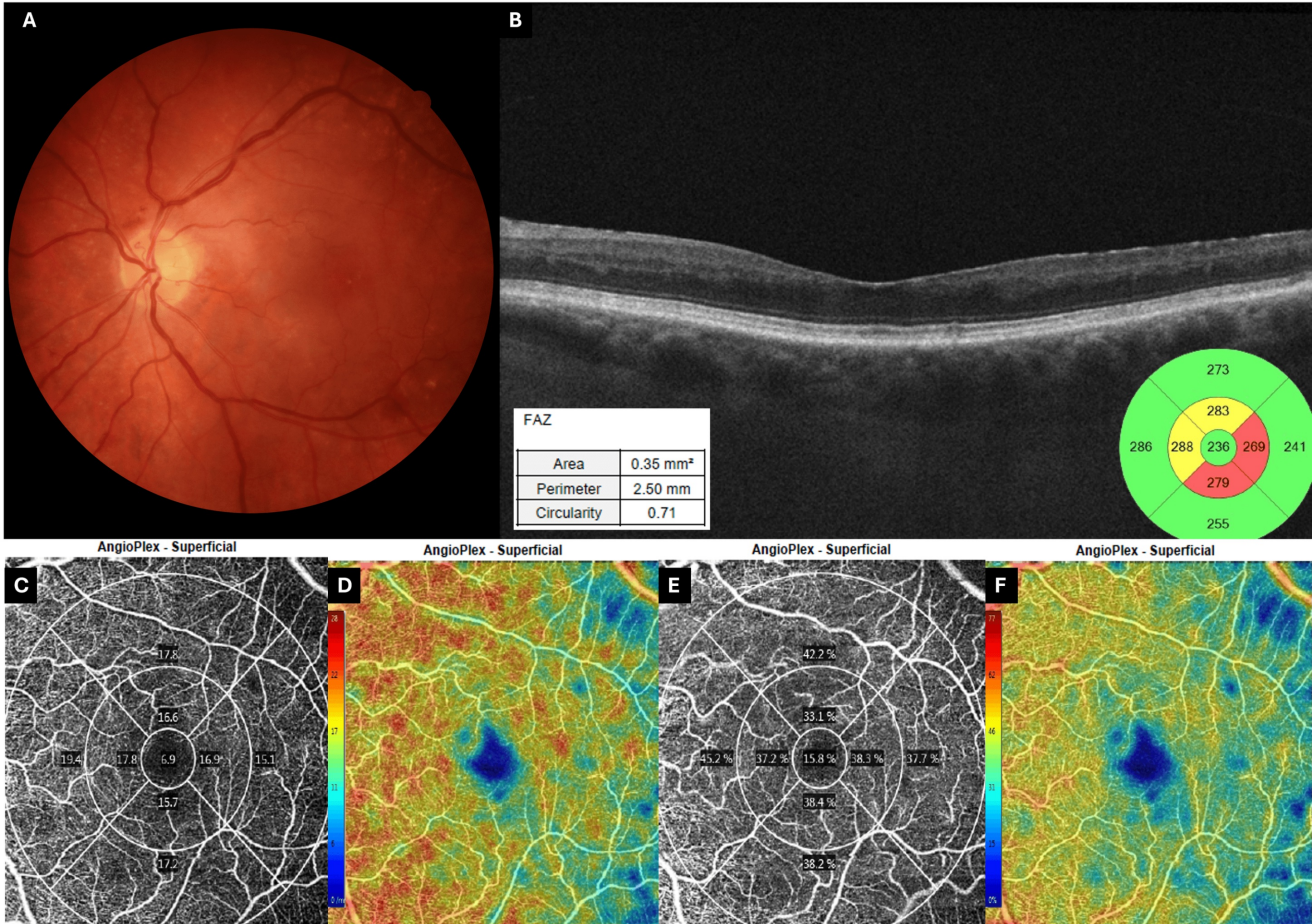
Three-month follow-up imaging of the affected (left) eye (A) Color fundus photograph demonstrates a quiescent posterior pole without new hemorrhage; the macula appears flattened and slightly pale. (B) Spectral-domain OCT reveals marked inner-retinal thinning (foveal thickness 236 µm). FAZ is closer to fellow eye values, though still irregular (0.35 mm², circularity 0.71), reflecting permanent capillary loss. (C, E) ETDRS vessel- and perfusion-density plots (respectively) show further decline in central macular flow (6.9%/15.8%), the lowest values recorded. (D, F) Corresponding color-coded deviation maps highlight an irregular blue hypoperfused core, corresponding to FAZ, bordered by relatively preserved, but still comparatively reduced, perifoveal circulation (scale ranging from blue/low to red/high density). OCT and OCT-A (6 × 6 mm) images acquired using Zeiss Cirrus 5000 OCT (Carl Zeiss Meditec AG, Baden-Württemberg, Germany). Fundus images acquired using Canon CR2 Digital Fundus Camera (Canon Inc., Ōta, Tokyo). OCT: optical coherence tomography, FAZ: foveal avascular zone, ETDRS: early treatment of diabetic retinopathy study

At the three-month follow-up, the patient's vision remained poor. Acute fundus findings had almost disappeared (Figure 10A). Extensive thinning of the macular area was noted, sparing the nerve fiber layer and ganglion cell layer (Figure 10B). OCT showed macular thinning corresponding to the affected layers. OCT-A vascular density and perfusion remained low (Figure 10C-10F).

**Figure 11:**
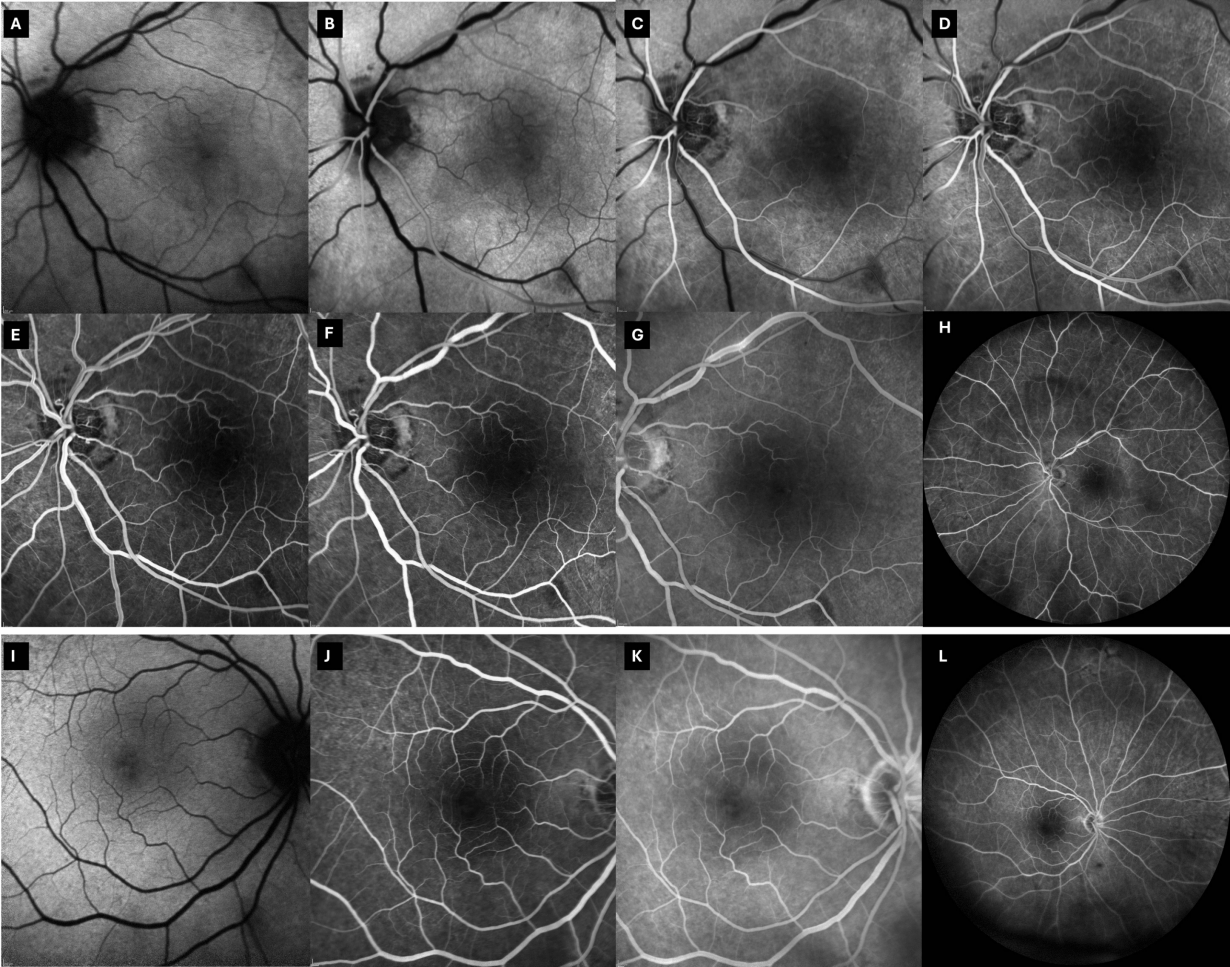
Three-month follow-up FAF and FA (A) FAF (left eye) shows macular hypo-FAF, likely reflecting regional non-perfusion–related signal differences and/or residual attenuation, rather than primary RPE atrophy. (B–D) Early arterial through early venous frames reveal normal arteriolar–venous transit without the segmental venous delay seen acutely. (E, F) Mid-phase FA demonstrates an irregular FAZ with persistent parafoveal capillary dropout and a hypofluorescent macular core relative to the fellow eye but no leakage. (G) Late FA confirms vessel patency with stable non-perfusion of the macular capillary bed. (H) Wide-field late frame shows a well-perfused retinal periphery, excluding chronic ischemia. (I–L) Control right eye: FAF and FA are entirely normal. Images acquired using Heidelberg SPECTRALIS (Heidelberg Engineering GmbH, Heidelberg, Germany). FAF: fundus autofluorescence, FA: fundus fluorescein angiography, FAZ: foveal avascular zone, RPE: retinal pigment epithelium

RAPD was still present, although diminished. At this time, a new FAF and FA were performed (Figure 11). FAF demonstrated macular hypoautofluorescence compared to the fellow eye. FAZ irregularity (though diminished), persistent macular capillary dropout, and a hypofluorescent macular core relative to the fellow eye were also observed. The right eye remained normal.

**Figure 12:**
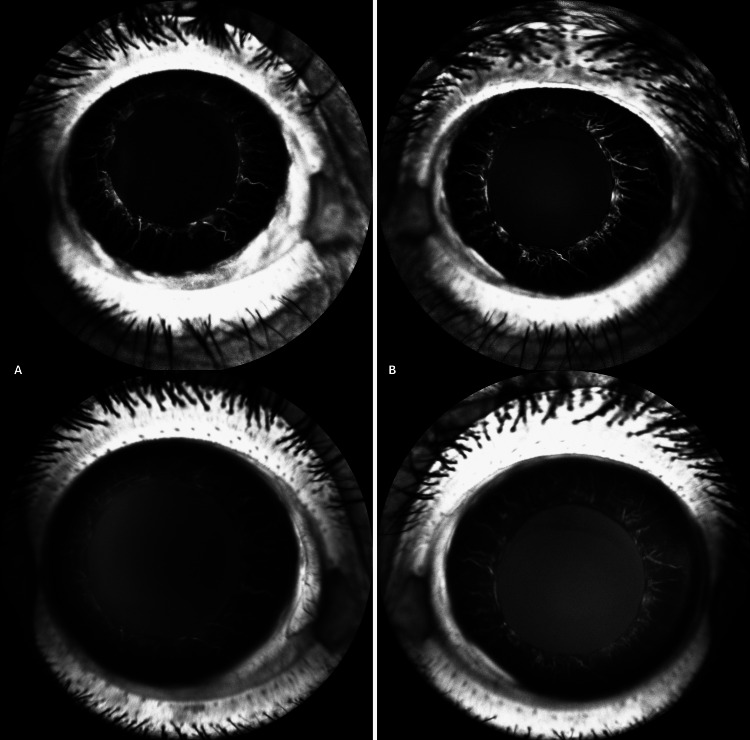
Three-month anterior-segment FA Column A, right (control) eye. Column B, left (affected) eye. Top panels: early phase. Bottom panels: late phase. In both eyes, iris vessels remain sharply outlined without diffuse hyperfluorescence or neovascular fronds, indicating that the pronounced iris leakage seen at 48 hours of FA in the left eye has resolved. Images acquired using Heidelberg SPECTRALIS (Heidelberg Engineering GmbH, Heidelberg, Germany). FA: fundus fluorescein angiography, VEGF: vascular endothelial growth factor

This time, the iris of the left eye was normal, without diffuse leakage (Figure 12).

Unfortunately, visual field testing was not feasible due to the severity of vision loss, with VA limited to counting fingers in the affected eye throughout follow-up. Confrontation fields showed a relative superonasal defect, supportive only, consistent with the greater inferotemporal macular atrophy on OCT. Microperimetry was not available in our clinic.

No macular edema or neovascularization has developed to prompt intervention with the use of panretinal or sectoral photocoagulation or the use of anti-vascular endothelial growth factors (VEGF). The patient remains under close ophthalmologic follow-up.

## Discussion

This case presents a striking clinical paradox: acute, profound monocular vision loss in the presence of relatively modest fundoscopic findings. Initial impressions suggested a possible transient or partial CRVO, but vision loss was disproportionate to the retinal findings. These inconsistencies prompted a deeper investigation through multimodal retinal imaging and systemic evaluation.

Multimodal imaging did not fully support the abovementioned interpretation: CRVO findings do exist but are surprisingly sparse and cannot explain symptoms adequately; OCT and OCT-A showed PAMM with INL hyperreflectivity with a fern-like pattern, which became more extensive on day-2 follow-up, and eventually significant inner retinal atrophy at the macula; FAF showed diffuse hypoautofluorescence of affected areas; FA demonstrated delayed arterial and venous filling with macular capillary drop-out with an enlarged and irregular FAZ; a RAPD was observed at 48 hours and persisted on follow-ups, indicating significant inner retinal dysfunction. Together, these findings, coupled with a newly discovered atrial fibrillation diagnosis, point to an acute ischemic event, most likely embolic in origin, with possible secondary hemodynamic effects, including venous outflow disturbance.

The diagnosis of previously undetected atrial fibrillation during systemic work-up provided a critical clue to the etiology of the retinal ischemic event. Atrial fibrillation is a well-established cause of thromboembolic phenomena, mostly involving the central nervous system, and is potentially underreported in retinal microvascular events. This is particularly true in elderly patients and may result in the release of both large and small emboli capable of reaching the ophthalmic circulation [3].

We propose that a microembolic event from undiagnosed atrial fibrillation caused acute ischemic injury at the capillary level, with secondary or concurrent hemodynamic compromise consistent with a transient or partial CRVO. The imaging and clinical findings support embolic occlusion of small-caliber retinal vessels, with possible secondary venous congestion contributing to the hemorrhagic changes. This microvascular compromise, localized to the macula, was sufficient to cause irreversible retinal ischemia and profound, persistent visual loss, even in the absence of classical signs of central retinal artery occlusion (CRAO) or CRVO.

It is well established that PAMM reflects ischemia at the level of the intermediate capillary plexus (ICP), particularly affecting the INL. It has been documented in a variety of retinal ischemic, surgical, and inflammatory conditions [2].

The pathophysiology of PAMM is still not clear. There is a consensus that PAMM appears due to sublethal ischemic hypoxia of the middle retinal tissue [2]. The outer plexiform layer and the INL are supplied with oxygen by both the retinal and choroidal circulation (watershed zones). In cases of reduced blood flow, even momentarily, these layers are the most sensitive to the reduced supply of oxygen [5]. Rapid return to normal perfusion might also play a role in the pathophysiology of PAMM due to a rapid increase in reactive oxygen species, nitric oxide neurotoxicity, inflammation, and extravascular leakage [2]. This could be explained by the metrics of OCT-A on day 2.

Patients with PAMM typically report subtle paracentral scotomas and mild visual disturbances [2]. In contrast, our patient presented with acute, severe vision loss. The discordance between mild retinal findings and profound vision loss, along with RAPD, prompted further ocular imaging and multisystemic investigation. Multimodal imaging and systemic findings supported a more serious microvascular insult involving the macular capillaries.

On en face OCT and OCT-A, three patterns of PAMM distribution have been observed. All patterns appear hyperreflective but correlate to different areas and specific mechanisms of microvascular ischemia. First, the arteriolar pattern follows the distribution of a sectorial artery occlusion [2]. Second, the globular pattern is described as one or multiple ovoid hyperreflective patches, possibly due to precapillary arterial occlusion [2,6]. Third, the fern-like pattern, as in our case, may be observed around the retinal venules due to stasis and reduced oxygenation of retinal tissue [2,6]. This third pattern demonstrates a spectrum of gradient and variation depending on the severity of ischemia [2]. It has been reported in patients with atrial fibrillation [3], suggesting that perivenular fern-like PAMM can appear in both embolic and venous outflow compromise [3].

FAF imaging in our case revealed pronounced hypoautofluorescence corresponding to areas of PAMM. Although PAMM lesions are usually subtle or undetectable on FAF [2,7], in this case, they were striking, likely due to significant INL edema due to extensive microvascular insult. This, in turn, masked the underlying retinal pigment epithelium (RPE) autofluorescence. Similar FAF patterns have been rarely described in severe microvascular insult with the retinal vein component [8]. The three-month FAF hypoautofluorescence likely reflects residual attenuation/non-perfusion effects, a conclusion supported by the preserved outer retina/RPE on OCT.

FA cannot distinguish between the ICP and the deep capillary plexus. It can, however, offer valuable insights into the state of the superficial vascular network and the underlying etiology, particularly when vascular compromise is a possibility [2]. The FA findings we described have been reported in cases of PAMM.

The presence of a RAPD at both one- and three-month follow-ups is particularly telling. RAPD is uncommon in non-ischemic CRVO [5] but consistent with severe inner retinal ischemia and dysfunction [9,10]. Given the absence of disc pallor or advanced optic neuropathy, we interpret the RAPD as an additional functional marker of deep retinal ischemia.

The worsening of flame-shaped hemorrhages at the optic disc can be explained by secondary venous congestion resulting from downstream capillary occlusion and partial or transient CRVO. Ischemia-induced VEGF release could have further increased vascular permeability and fragility. Peripheral hemorrhages are consistent with secondary venous stasis, supporting the partial CRVO component of the overall ischemic mechanism.

While our case presented some overlapping features with CRVO-associated PAMM, its clinical course was atypical. Visual prognosis in CRVO-associated PAMM varies depending on the pattern observed. At final follow-up, the mean VA ranged from 20/43-20/120 to 20/25-20/42 Snellen [3,4,11,12]. Eyes with perivenular fern-like PAMM had the best prognosis for VA compared to eyes with arteriolar or globular PAMM [3]. This was not observed in our case, where VA remained poor. This disparity in visual outcome suggests that in our patient, PAMM likely resulted from concurrent, more severe microvascular ischemia rather than mild venous congestion alone, consistent with a primary microembolic mechanism.

Human data on very early iris leakage are limited. However, classic fluorescein angiography series have already demonstrated that diffuse iris vessel staining can appear within days of an occlusive event and resolve in later stages [13,14]. These findings, taken together with the scarcity of CRVO findings in the patient, propose a more complex microvascular event with elements of venous stasis. Additionally, these models support our hypothesis that late iris hyperfluorescence, in the acute phase of the occlusive episode, could be attributed to the release of VEGF and other cytokines from the ischemic posterior pole. These cytokines can pass from the posterior to the anterior segment, especially in eyes without an intact crystalline lens (e.g., eyes that have undergone phacoemulsification), as in our patient, a conclusion supported by animal model studies [15]. This speculation is strengthened by the fact that iris hyperfluorescence was present on the day-2 FA and absent at the three-month follow-up.

Quantitative OCT-A metrics of our patient are summarized in Table 1.

**Table 1:** Summary of OCT-A metrics of the patient presented in the report at various follow-ups Except for the central ring, the first and second ring values are averaged from the corresponding ETDRS OCT-A grid quadrants. OCT-A: optical coherence tomography-angiography, FAZ: foveal avascular zone, VD: vessel density, PD: perfusion density, ETDRS: early treatment of diabetic retinopathy study

Timepoint	FAZ area (mm²)	Perimeter (mm)	Circularity	Central VD	Central PD	1st ring VD	1st ring PD	2nd ring VD	2nd ring PD
At presentation	0.43	3.07	0.57	7.50%	15.80%	15.90%	36.90%	18.90%	44.10%
Day 2	0.18	1.77	0.72	15.40%	33.20%	19.00%	45.90%	18.90%	46.20%
1 month	0.46	3.2	0.57	8.60%	17.80%	16.20%	39.50%	15.70%	39.60%
3 months	0.35	2.5	0.71	6.90%	15.80%	15.70%	38.00%	16.70%	40.80%
Normal eye	0.34	2.3	0.81	12.40%	27.90%	19.00%	45.60%	18.70%	46.80%

The findings could support a hypothesis of transient vascular compromise with reperfusion [16]. However, it is more likely that VEGF overexpression due to macular ischemia led to a transient increase in vascular density and perfusion metrics due to capillary dilation, which ceased after chronicity ensued, especially when compared to OCT-A metrics of the fellow eye [17]. All images were acquired using eye tracking. All images had a signal strength of at least 9 out of 10. At least two different image acquisitions were performed at each follow-up. All values were computed with the device's default analysis, and results were corroborated from each subsequent acquisition. Segmentation was done automatically, and all images were checked for segmentation errors. Images with severe segmentation errors and motion artifacts were discarded.

Differential diagnosis in cases of acute vision loss with similar findings should include isolated non-ischemic CRVO (ruled out in our case due to minimal retinal findings with severe vision loss and clinical course of patient), ischemic CRVO (ruled out due to mild fundus findings and absent posterior segment ischemia other than macular region), isolated PAMM (ruled out due to clinical findings and severe vision loss without improvement), classic CRAO (no cherry-red spot, no boxcarring vessels, no emboli found, no retinal whitening), acute macular neuroretinopathy (excluded due to patient age, absence of wedge-shaped parafoveal lesions and characteristic OCT findings), non-arteritic anterior ischemic optic neuropathy (explains RAPD but no true disc edema present, optic disc remained normal through follow-ups, macular-based ischemia), GCA (explains profound vision loss but ruled out due to lab results and absence of relevant symptoms) and other autoimmune or infectious vascular diseases, specific to patient profile (ruled out due to lab results) [2].

Additionally, worthy of note is the probability of a short posterior ciliary artery embolus with partial/transient CRVO (although indocyanine green angiography was not performed, this possibility is highly unlikely as no Amalric sign was present, the early choroidal phase on FA was normal, the outer retina/RPE remained intact, the optic nerve head structure remained sound, and the RNFL/ganglion cell layer remained normal). All the abovementioned diagnoses are either excluded outright or don’t fit adequately with the presented findings and clinical course. In this case, no true intervention can be made as of the writing of this report, other than the control of systemic risk factors coupled with close ophthalmologic observation.

## Conclusions

We presented a rare presentation of acute, profound vision loss due to retinal capillary ischemia in a patient with newly diagnosed atrial fibrillation. Multimodal imaging revealed inner retinal infarction and perfusion loss consistent with PAMM, alongside delayed vascular filling and optic disc leakage, suggesting both ischemia and hemodynamic disruption. The pattern of ischemia, clinical course, and newly diagnosed atrial fibrillation strongly support a primary microembolic mechanism with concurrent transient venous outflow impairment, consistent with a partial CRVO component. This case highlights the importance of considering systemic embolic etiologies in unexplained visual loss, especially when retinal findings are scarce, and demonstrates the utility of multimodal imaging coupled with a prompt multidisciplinary approach in detecting subtle but functionally devastating ischemic insults.

Our case report has some limitations. No embolus was directly visualized, and early-phase FA was delayed. However, the convergence of imaging, clinical signs, and the newly diagnosed atrial fibrillation provides a compelling framework for the underlying mechanism. Further studies are needed to characterize such presentations better and evaluate their frequency in systemic cardioembolic conditions, which are underrepresented and possibly underdiagnosed.

## Appendices

**Table 2:** Tests ordered for patient at presentation and at the two-day follow-up * lab tests ordered at presentation WBC: white blood cell count, MCV: mean corpuscular volume, ESR: erythrocyte sedimentation rate, CRP: C-reactive protein, HbA1c: hemoglobin A1c (glycated hemoglobin), CD4+/CD8+: cluster of differentiation 4/cluster of differentiation 8, AST: aspartate aminotransferase, ALT: alanine aminotransferase, ALP: alkaline phosphatase, PT: prothrombin time, INR: international normalized ratio, aPTT: activated partial thromboplastin time, TSH: thyroid-stimulating hormone, LDL: low-density lipoprotein cholesterol, HDL: high-density lipoprotein cholesterol, RPR: rapid plasma reagin, VDRL: venereal disease research laboratory test, EIA: enzyme immunoassay, TP-PA: treponema pallidum particle agglutination assay, ANA: antinuclear antibody, ANCA: antineutrophil cytoplasmic antibody, IgG/IgM: immunoglobulin G/immunoglobulin M, Na⁺: sodium, K⁺: potassium, Ca: calcium

Tests	Results	Tests	Results
WBC*	6.87 * 10^3/ μL	AST*	26 U/L
Hemoglobin*	13.7 g/dL	ALT*	20 U/L
Hematocrit*	43.50%	ALP	70 U/L
MCV*	95 fL	PT*	13 s
Platelets*	277×10^3^/μL	INR*	1.1
ESR *	31 mm/h	aPTT*	30 s
CRP*	5 mg/L	Fibrinogen*	310 mg/dL
Fasting plasma glucose*	93 mg/dL	TSH	4,35 μIU/L
HbA1c	5,2 %	Vitamin B₁₂	600 pg/mL
Total cholesterol	189 mg/dl	Folate (serum)	8 ng/mL
LDL	87 mg/dl	Homocysteine	10 µmol/L
HDL	82 mg/dL	Serum protein electrophoresis	No monoclonal spike
Triglycerides	97 mg/dL	Syphilis serology (RPR/VDRL and treponemal EIA/TP-PA)	Non-reactive/negative
Creatinine*	0.96 mg/dL	Lupus anticoagulant	Negative
Uric acid	6.2 mg/dl	Anticardiolipin IgG/IgM	Negative
Urea*	68 mg/dL	Anti-β₂-glycoprotein I IgG/IgM	Negative
Total blood proteins	7.7 g/dl	ANA	Negative
Albumin	4.3 g/dl	ANCA	Negative
Na+*	140 mEq/l	Rheumatoid factor	<20 IU/mL
K+*	3.77 mEq/l	Flow cytometry-peripheral blood	CD4+/CD8+ = 2.1
Ca	9.2 mg/dl		
